# COPD is associated with poorer survival in patients with advanced non-small cell lung cancer treated with first-line ICI plus chemotherapy

**DOI:** 10.1186/s12890-026-04509-3

**Published:** 2026-07-20

**Authors:** Akihisa Takano, Daisuke Shibahara, Hiroaki Ogata, Sai Yasukochi, Ryosuke Matsukane, Toshifumi Ninomiya, Takahiro Utsumi, Yuki Ikematsu, Kohei Otsubo, Yoshimasa Shiraishi, Eiji Iwama, Mayako Uchida, Isamu Okamoto

**Affiliations:** 1 Department of Respiratory Medicine, Graduate School of Medical Sciences, Kyushu University, 3-1-1 Maidashi, Higashi-ku, Fukuoka, 812-8582 Japan; 2 Department of Pharmacy, Kyushu University Hospital, Fukuoka, Japan

**Keywords:** Chronic obstructive pulmonary disease, Non-small cell lung cancer, Immune checkpoint inhibitors, Chemotherapy, Progression-free survival, Overall survival

## Abstract

**Background:**

Chronic obstructive pulmonary disease (COPD) frequently coexists with non–small cell lung cancer (NSCLC). Previous studies suggest that COPD may be associated with improved outcomes in patients receiving immune checkpoint inhibitor (ICI) monotherapy, but its impact in patients treated with ICI plus chemotherapy remains unclear.

**Methods:**

In this retrospective single-institution study, we evaluated 55 ever-smokers with stage IV NSCLC treated with first-line ICI plus chemotherapy who underwent pulmonary function testing. COPD was defined as a post-bronchodilator FEV_1_/FVC ratio < 0.70, and patients were classified into COPD (*n* = 33) and non-COPD (*n* = 22) groups. Progression-free survival (PFS) and overall survival (OS) were analyzed using Kaplan–Meier methods and Cox proportional hazards models. To reduce baseline confounding, inverse probability of treatment weighting (IPTW) based on propensity scores was applied.

**Results:**

In unadjusted analyses, median PFS was 5.3 months in the COPD group and 11.1 months in the non-COPD group (hazard ratio [HR] 1.79, 95% confidence interval [CI] 0.95–3.37; *p* = 0.071). After IPTW adjustment, PFS was significantly shorter in the COPD group (median 4.6 months vs 13.7 months; HR 2.54, 95% CI 1.29–5.00; *p* = 0.007). In unadjusted analyses, median OS was 12.7 months in the COPD group and not reached in the non-COPD group (HR 1.56, 95% CI 0.73–3.33; *p* = 0.247). After IPTW adjustment, OS was significantly shorter in the COPD group (median 11.8 months vs not reached; HR 3.00, 95% CI 1.16–7.73; *p* = 0.023).

**Conclusions:**

COPD may be associated with poorer survival among ever-smokers with advanced NSCLC receiving first-line ICI plus chemotherapy, although further validation in larger cohorts is warranted.

**Supplementary Information:**

The online version contains supplementary material available at https://doi.org/10.1186/s12890-026-04509-3.

## Background

Chronic obstructive pulmonary disease (COPD) is a common comorbidity among patients with non-small cell lung cancer (NSCLC), as both conditions share cigarette smoking as a major risk factor [1–3]. COPD is characterized by persistent airflow limitation and chronic airway inflammation, and is also associated with systemic inflammatory responses and immune dysregulation, which may influence tumor biology and treatment response in lung cancer [2, 3]. Immune checkpoint inhibitors (ICIs), including anti-programmed death-1 (PD-1) antibodies, have become a cornerstone of first-line therapy for advanced NSCLC. In combination with platinum-based chemotherapy, ICIs are now widely used as a standard first-line treatment [4, 5].

Previous studies have reported that COPD may be associated with enhanced responsiveness to ICI monotherapy [6–11]. However, the clinical impact of COPD among patients receiving first-line ICI plus chemotherapy has not been well characterized. Therefore, we conducted a retrospective study to evaluate the prognostic impact of COPD on progression-free survival (PFS) and overall survival (OS) among ever-smokers with stage IV NSCLC treated with first-line ICI plus chemotherapy. To address potential baseline confounding between patients with and without COPD, inverse probability of treatment weighting (IPTW) was applied, and survival outcomes were evaluated in both unadjusted and weighted analyses.

## Methods

### Study design and patients

This retrospective single-institution study included patients with stage IV NSCLC who received first-line ICI plus chemotherapy at Kyushu University Hospital between January 2016 and May 2024. Eligibility required a history of smoking and available pulmonary function test (PFT) data; never-smokers and patients without PFT results were excluded. COPD was defined according to the Global Initiative for Chronic Obstructive Lung Disease (GOLD) criteria as a post-bronchodilator forced expiratory volume in one second to forced vital capacity ratio (FEV_1_/FVC) < 0.70. Patients were classified into COPD and non-COPD groups based on spirometric findings.

### Data collection

Clinical data were extracted from electronic medical records, including age, sex, smoking history, histology, PD-L1 tumor proportion score (TPS), Eastern Cooperative Oncology Group performance status (ECOG PS), spirometric parameters, driver alteration status, COPD severity (GOLD classification), laboratory findings [albumin, C-reactive protein (CRP), and neutrophil-to-lymphocyte ratio (NLR)], body mass index (BMI), comorbidities, radiological findings, treatment regimens, treatment-related adverse events, and treatment modifications. The presence of emphysema and interstitial lung disease (ILD) was determined based on chest CT reports documented in the electronic medical records.

### Study outcomes

The primary outcome was PFS, defined as the time from initiation of first-line ICI plus chemotherapy to disease progression or death from any cause. The secondary outcome was OS, defined as the time from treatment initiation to death from any cause. Patients without documented events were censored at the date of last follow-up.

### Statistical analysis

Baseline characteristics were compared between the COPD and non-COPD groups. Categorical variables were analyzed using the chi-square test or Fisher’s exact test, as appropriate, and continuous variables using the Student’s t-test or Wilcoxon rank-sum test according to the distribution. PFS and OS were estimated using the Kaplan–Meier method. Comparisons between the COPD and non-COPD groups were performed using Cox proportional hazards regression models, and hazard ratios (HR) with 95% confidence intervals (CI) were calculated. To reduce baseline confounding, IPTW based on propensity scores was applied. Propensity scores were estimated using a logistic regression model including age, sex, smoking exposure (pack-years), histology (squamous vs. non-squamous), PD-L1 TPS (TPS; 0%, 1–49%, and 50–100%), and ECOG PS (0 vs. 1). Patients with missing PD-L1 TPS were excluded from propensity score estimation and IPTW-adjusted analyses. Weighted Cox proportional hazards models were used for IPTW-adjusted analyses. Covariate balance before and after weighting was assessed using standardized mean differences (SMDs), with values < 0.10 considered indicative of adequate balance. All analyses were performed using R software (version 4.3.0 or later). A two-sided *p*-value < 0.05 was considered statistically significant.

### Sensitivity analysis

As a sensitivity analysis, propensity scores were re-estimated after additionally incorporating emphysema status and driver alteration status (present vs. absent/unknown) into the primary propensity score model. Covariate balance, PFS, and OS were evaluated in the same manner as in the primary analysis.

## Results

### Patient selection and baseline characteristics

**Fig. 1:**
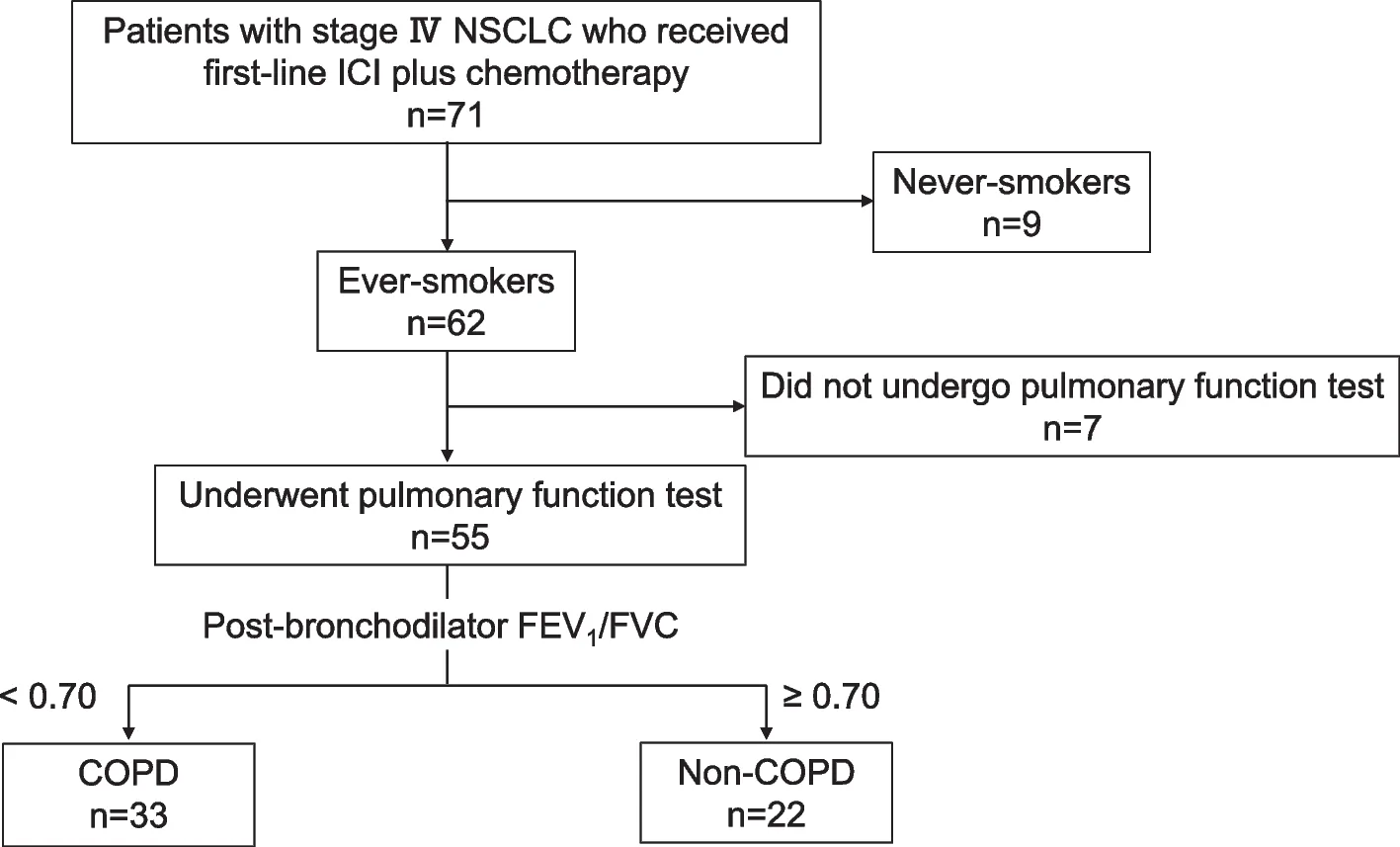
Patient selection flow diagram. Flow diagram illustrating patient selection and classification based on smoking history and post-bronchodilator spirometry. Abbreviations: NSCLC, non-small cell lung cancer; ICI, immune checkpoint inhibitor; COPD, chronic obstructive pulmonary disease; FEV_1_, forced expiratory volume in 1 s; FVC, forced vital capacity

During the study period, 71 patients with stage IV NSCLC received first-line ICI plus chemotherapy. Nine never-smokers were excluded, and among the remaining ever-smokers, seven patients without available pulmonary function test data were further excluded. The final study cohort comprised 55 ever-smokers with available PFT data (Fig. 1). Based on post-bronchodilator spirometry, patients were classified into a COPD group (*n* = 33) and a non-COPD group (*n* = 22).

**Fig. 2:**
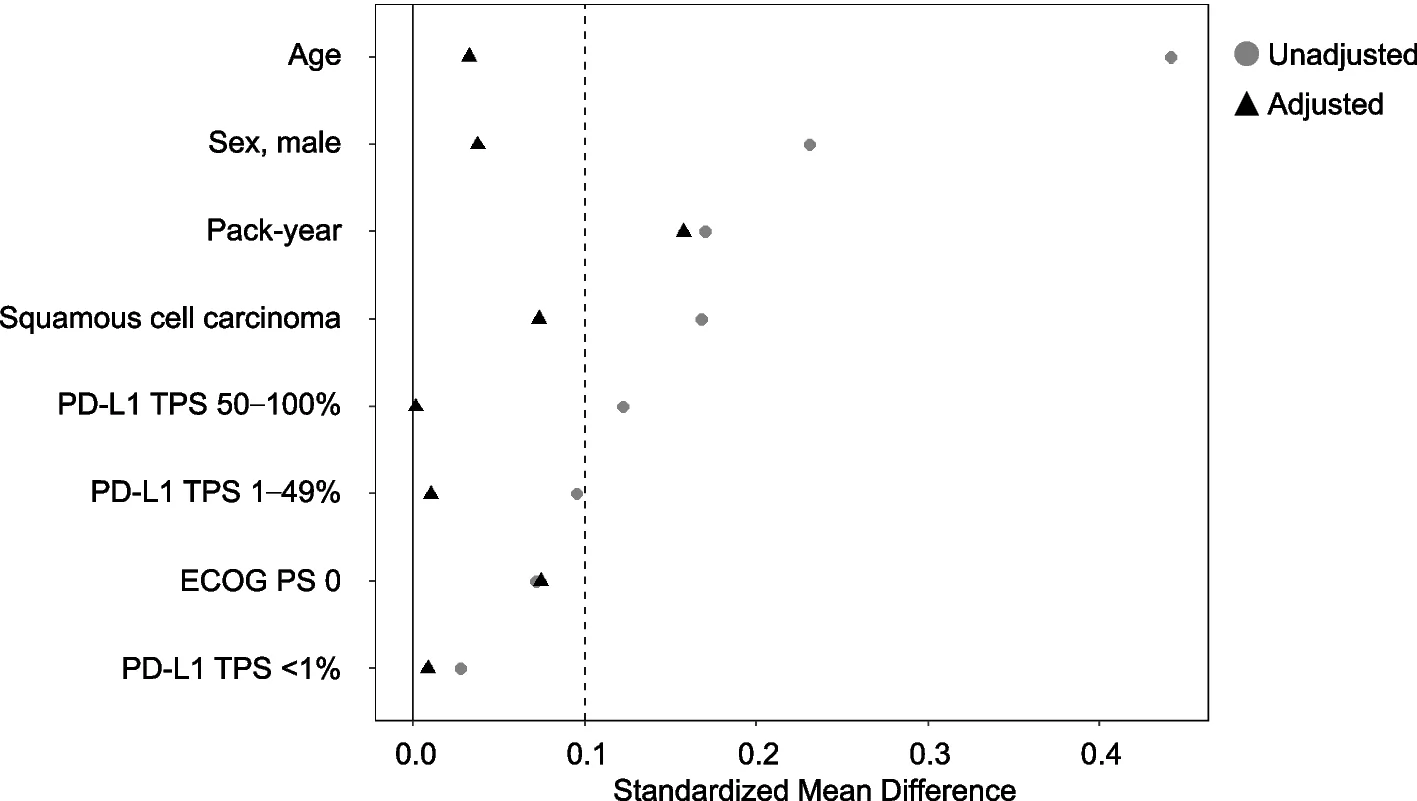
Covariate balance before and after IPTW adjustment. Standardized mean differences (SMDs) for baseline covariates before and after inverse probability of treatment weighting (IPTW) between the COPD and non-COPD groups. The dashed line indicates an absolute SMD of 0.10. Abbreviations: IPTW, inverse probability of treatment weighting; COPD, chronic obstructive pulmonary disease; PD-L1, programmed death-ligand 1; TPS, tumor proportion score; ECOG PS, Eastern Cooperative Oncology Group performance status; SMD, standardized mean difference

Baseline characteristics before and after IPTW adjustment are summarized in Table 1. In the unadjusted cohort, patients with COPD were more frequently male than those without COPD (97.0% vs. 77.3%, *p* = 0.064). A modest imbalance was also observed for age and tumor histology. Other baseline variables, including smoking exposure, PD-L1 TPS, and ECOG PS, were generally comparable between the two groups. Among the 55 patients in the primary cohort, five patients with missing PD-L1 TPS were excluded from IPTW-adjusted analyses. IPTW was therefore applied to the remaining 50 patients with complete covariate data. After weighting, baseline characteristics were more evenly distributed between the COPD and non-COPD groups (Table 1). Imbalances observed in the unadjusted cohort were attenuated, as reflected by reduced SMDs. Most covariates achieved an absolute SMD < 0.10, indicating adequate covariate balance in the weighted cohort (Fig. 2).

**Table 1:** Baseline characteristics of the COPD and non-COPD groups before and after IPTW adjustment

	**Unadjusted**			**IPTW adjusted**		
	**COPD**	**Non-COPD**	**P**	**COPD**	**Non-COPD**	**SMD**
	**(*n* = 33)**	**(*n* = 22)**				
Age (years), mean (SD)	64.9 (7.9)	62.7 (10.8)	0.386	63.4 (8.1)	63.1 (10.3)	0.033
Sex, male (%)	32 (97.0)	17 (77.3)	0.064	91.0	87.3	0.037
Pack-year, median (IQR)	48.0 (34.5–58.0)	38.5 (25.1–55.1)	0.200	46.4 (35.4–58.7)	41.0 (31.1–68.8)	0.158
Histology (%)			0.196			0.073
Squamous cell carcinoma	16 (48.5)	6 (27.3)		43.6	36.3	
Non-squamous cell carcinoma	17 (51.5)	16 (72.7)		56.4	63.7	
PD-L1 TPS (%)						
< 1	9 (27.3)	5 (22.7)	0.950	28.6	27.8	0.009
1–49	16 (48.5)	8 (36.4)	0.542	48.3	49.3	0.010
50–100	6 (18.2)	6 (27.3)	0.641	23.1	22.9	0.002
^*^Not available	2 (6.1)	3 (13.6)	0.632			
ECOG PS (%)						0.074
0	14 (42.4)	6 (27.3)	0.391	37.0	29.6	
1	19 (57.6)	16 (72.7)	0.391	63.0	70.4	
≥ 2	0 (0.0)	0 (0.0)	1.000			

IPTW was based on propensity scores estimated using age, sex, pack-year, histology, PD-L1 TPS, and ECOG PS

*Abbreviations*: *COPD* chronic obstructive pulmonary disease, *IPTW* inverse probability of treatment weighting, *SD* standard deviation, *IQR* interquartile range, *PD-L1* programmed death-ligand 1, *TPS* tumor proportion score, *ECOG PS* Eastern Cooperative Oncology Group performance status, *SMD* standardized mean difference

^*^Five patients with missing PD-L1 TPS were excluded from IPTW-adjusted analyses. Values are presented as n (%) for unadjusted data and weighted percentages for IPTW-adjusted data, unless otherwise indicated

Additional baseline clinical characteristics not included in the IPTW model are summarized in Table 2. Driver alterations were numerically more frequent in the non-COPD group. Although albumin levels tended to be lower in the COPD group, CRP, NLR, and BMI were generally comparable. Comorbidities were also generally similar between the groups. As expected by the diagnostic definition, the FEV_1_/FVC ratio was significantly lower in the COPD group, and emphysema was more frequently observed radiologically, whereas interstitial lung disease was rare in both groups. Most patients with COPD had mild-to-moderate airflow limitation (GOLD stage I–II). Regarding treatment regimens, paclitaxel-based chemotherapy was more frequently administered in the COPD group, while the distribution of ICIs was comparable between the groups.

**Table 2:** Additional baseline clinical characteristics not included in the IPTW model

	**Unadjusted**		
	**COPD**	**Non-COPD**	**P**
	**(*n* = 33)**	**(*n* = 22)**	
Driver alteration status (%)			
EGFR/ALK	0 (0.0)	1 (4.5)	0.400
Others	3 (9.1)	4 (18.2)	0.419
Negative	28 (84.8)	16 (72.7)	0.316
Unknown	2 (6.1)	1 (4.5)	1.000
Laboratory findings, median (IQR)			
Albumin, g/dL	3.40 (3.10–3.70)	3.80 (3.23–4.00)	0.054
CRP, mg/dL	1.04 (0.63–4.13)	1.70 (0.19–4.21)	0.744
NLR	4.05 (2.92–4.94)	3.82 (2.86–6.74)	0.689
BMI, median (IQR)	21.75 (20.56–24.01)	21.55 (18.58–23.96)	0.445
Comorbidities (%)			
Diabetes mellitus	4 (12.1)	4 (18.2)	0.700
Cardiovascular disease	6 (18.2)	7 (31.8)	0.334
History of other malignancy	1 (3.0)	4 (18.2)	0.145
FEV_1_/FVC ratio (%), median (IQR)	62.70 (54.40–64.90)	76.40 (71.98–79.10)	< 0.001
Radiological findings (%)			
Emphysema	27 (81.8)	12 (54.5)	0.038
Interstitial lung disease	0 (0.0)	1 (4.5)	0.400
GOLD stage (%)			
GOLD I	6 (18.2)	—	
GOLD II	25 (75.8)	—	
GOLD III	2 (6.1)	—	
GOLD IV	0 (0.0)	—	
Chemotherapy regimen (%)			0.098
CDDP/CBDCA + PEM	14 (42.4)	15 (68.2)	
CDDP/CBDCA + PTX/nab-PTX	19 (57.6)	7 (31.8)	
ICI (%)			0.753
Pembrolizumab	22 (66.7)	13 (59.1)	
Atezolizumab	2 (6.1)	2 (9.1)	
Nivolumab + Ipilimumab	9 (27.3)	7 (31.8)	

Others included KRAS and HER2 alterations

*Abbreviations*: *COPD* chronic obstructive pulmonary disease, *EGFR* epidermal growth factor receptor, *ALK* anaplastic lymphoma kinase, *IQR* interquartile range, *CRP* C-reactive protein, *NLR* neutrophil-to-lymphocyte ratio, *BMI* body mass index, *FEV*_*1*_ forced expiratory volume in 1 s, *FVC* forced vital capacity, *GOLD* Global Initiative for Chronic Obstructive Lung Disease, *CDDP* cisplatin, *CBDCA* carboplatin, *PEM* pemetrexed, *PTX* paclitaxel, *nab-PTX* nab-paclitaxel, *ICI* immune checkpoint inhibitor

Treatment-related adverse events and treatment modifications are summarized in Supplementary Table 1. Febrile neutropenia tended to occur more frequently in the non-COPD group, whereas pneumonia (Grade ≥ 2) and other infectious complications were more common in the COPD group. Although dose reduction tended to be more frequent in the non-COPD group, treatment discontinuation occurred only in the COPD group. Grade ≥ 3 immune-related adverse events were generally comparable between the groups (21.2% vs. 31.8%; *p* = 0.529).

### Progression-free survival

**Fig. 3:**
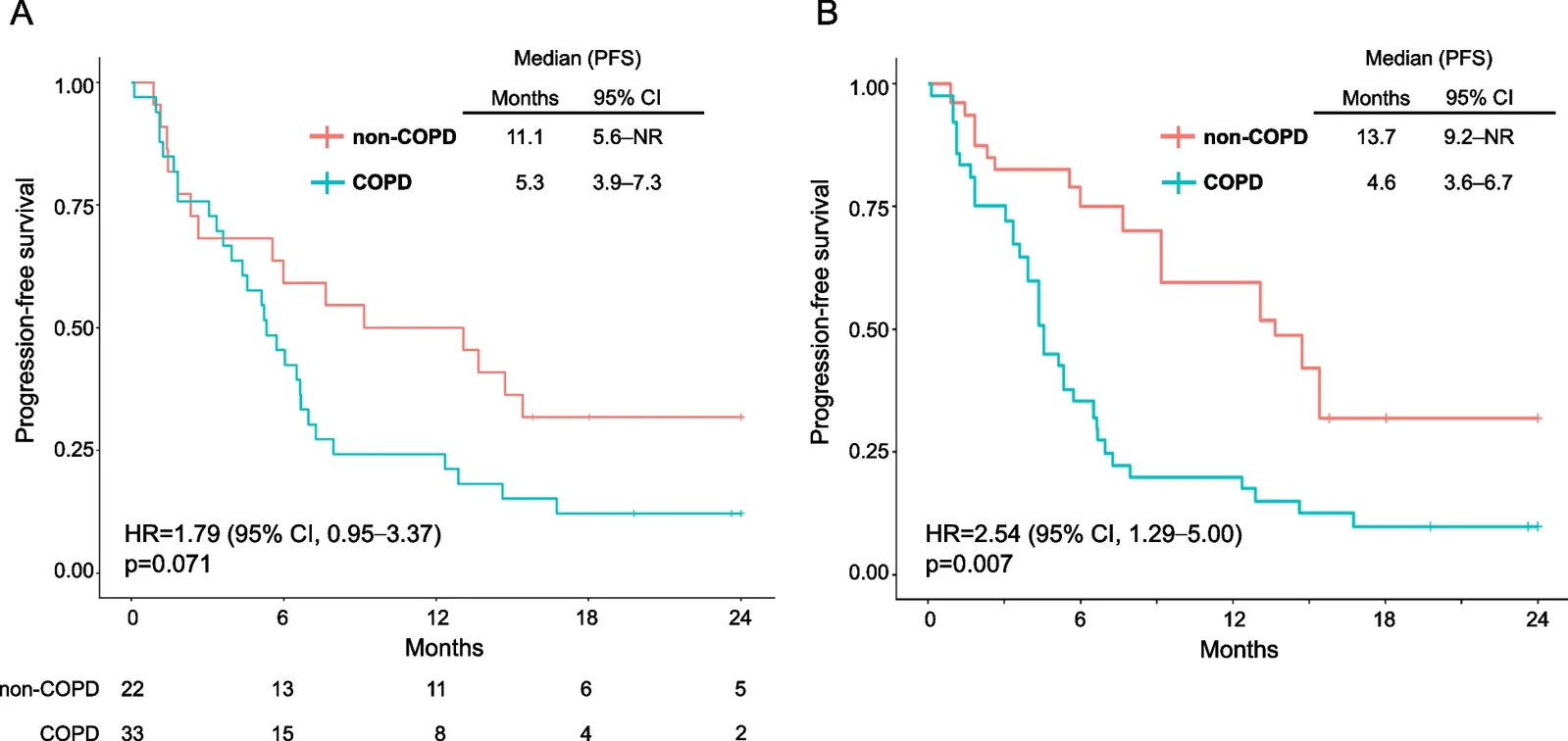
Progression-free survival according to COPD status. Kaplan–Meier curves for progression-free survival (PFS) in the COPD and non-COPD groups in the unadjusted cohort (**A**) and after inverse probability of treatment weighting (IPTW) (**B**). Abbreviations: COPD, chronic obstructive pulmonary disease; PFS, progression-free survival; IPTW, inverse probability of treatment weighting; HR, hazard ratio; CI, confidence interval; NR, not reached

Kaplan–Meier curves for PFS are shown in Fig. 3. In the unadjusted analysis (Fig. 3A), median PFS was 5.3 months (95% CI, 3.9–7.3 months) in the COPD group and 11.1 months (95% CI, 5.6 months–not reached) in the non-COPD group (HR, 1.79; 95% CI, 0.95–3.37; *p* = 0.071), suggesting a trend toward shorter PFS in the COPD group. After IPTW adjustment (Fig. 3B), PFS was significantly shorter in the COPD group (median, 4.6 months; 95% CI, 3.6–6.7 months) than in the non-COPD group (median, 13.7 months; 95% CI, 9.2 months–not reached) (HR, 2.54; 95% CI, 1.29–5.00; *p* = 0.007).

### Overall survival

**Fig. 4:**
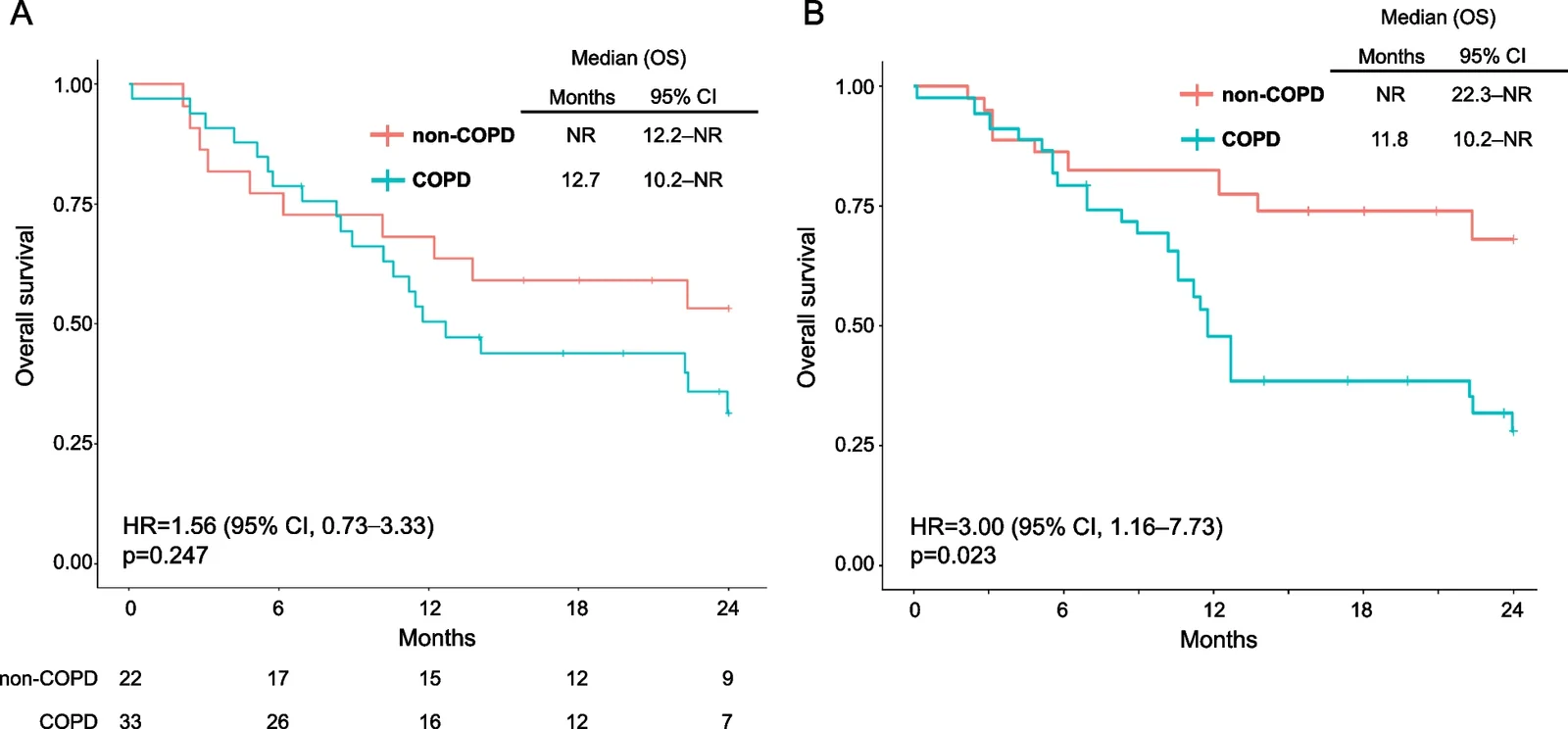
Overall survival according to COPD status. Kaplan–Meier curves for overall survival (OS) in the COPD and non-COPD groups in the unadjusted cohort (**A**) and after inverse probability of treatment weighting (IPTW) (**B**). Abbreviations: COPD, chronic obstructive pulmonary disease; OS, overall survival; IPTW, inverse probability of treatment weighting; HR, hazard ratio; CI, confidence interval; NR, not reached

Kaplan–Meier curves for OS are shown in Fig. 4. In the unadjusted analysis (Fig. 4A), median OS was 12.7 months (95% CI, 10.2 months–not reached) in the COPD group and not reached (95% CI, 12.2 months–not reached) in the non-COPD group (HR, 1.56, 95% CI, 0.73–3.33; *p* = 0.247). After IPTW adjustment (Fig. 4B), OS was significantly shorter in the COPD group (median, 11.8 months; 95% CI, 10.2 months–not reached) than in the non-COPD group (median not reached, 95% CI, 22.3 months–not reached) (HR, 3.00; 95% CI, 1.16–7.73; *p* = 0.023).

### Sensitivity analyses

In the primary IPTW analysis, COPD was associated with significantly shorter PFS and poorer OS. To assess the robustness of the primary findings, we performed an exploratory sensitivity analysis incorporating driver alteration status and emphysema status into the propensity score model. Adequate covariate balance was achieved after weighting (Supplementary Table 2 and Supplementary Fig. 1). The association between COPD and shorter PFS remained significant in the sensitivity analysis (Supplementary Fig. 2A). A similar trend toward poorer OS was observed, although statistical significance was not maintained (Supplementary Fig. 2B).

## Discussion

In this retrospective study, we evaluated the prognostic impact of COPD among ever-smokers with stage IV NSCLC treated with first-line ICI plus chemotherapy. Survival outcomes were consistently poorer in patients with COPD and became statistically significant after IPTW adjustment, suggesting a potential association between COPD and poorer survival in the setting of chemoimmunotherapy.

In the pre-ICI era, when platinum-based chemotherapy was the standard treatment for advanced NSCLC, COPD was consistently reported as an adverse prognostic factor [12–15]. This association has often been attributed to chronic airway inflammation and structural airway damage associated with COPD. These changes may increase susceptibility to respiratory complications and reduce tolerance to cytotoxic chemotherapy, thereby potentially contributing to poorer outcomes [2, 3].

In contrast, during the ICI era, most studies have reported that COPD was associated with improved responsiveness to ICI monotherapy, potentially reflecting smoking-related genomic instability, higher tumor mutational burden, and increased tumor-infiltrating lymphocytes [6, 7, 16, 17]. These observations raise the possibility that COPD-related inflammatory and immunologic alterations may enhance sensitivity to immune checkpoint blockade in certain contexts. However, it remains unclear whether the survival advantage associated with COPD in ICI monotherapy is maintained when chemotherapy is added. The addition of cytotoxic chemotherapy may alter this balance, as treatment-related toxicity and reduced treatment tolerance could offset this potential benefit. In our cohort, patients with COPD experienced significantly shorter PFS and OS than those without COPD after IPTW adjustment. Several mechanisms may account for this observation. Because chronic systemic inflammation and frailty-related conditions are frequently observed in patients with COPD [18–20] and have been associated with poorer outcomes in patients with lung cancer [21], we explored whether these factors contributed to the observed survival difference. Although albumin tended to be lower in patients with COPD, CRP, NLR, BMI, and major comorbidities were generally comparable between the groups. These findings suggest that the inflammatory and nutritional factors evaluated in this study may not fully explain the observed survival disadvantage. The relatively favorable baseline condition of our cohort, characterized by ECOG PS 0–1 in all patients and predominantly mild-to-moderate COPD (GOLD stage I–II), may have attenuated differences in frailty-related characteristics between the groups. Nevertheless, residual confounding by unmeasured factors, including cachexia, sarcopenia, symptom burden, and exacerbation history, cannot be excluded.

We next explored whether treatment-related factors may have contributed to the observed differences in outcome. Febrile neutropenia was more common in the non-COPD group, whereas patients with COPD experienced more pneumonia and other infectious complications. In addition, dose reduction was more frequent in the non-COPD group, while treatment discontinuation tended to occur more often in patients with COPD. These findings raise the possibility that COPD-related pulmonary vulnerability, rather than chemotherapy-related hematologic toxicity alone, may have contributed to poorer clinical outcomes. However, neither baseline clinical characteristics nor treatment-related events fully explained the observed survival disadvantage. Additional biological mechanisms associated with COPD may therefore contribute to outcomes in patients receiving chemoimmunotherapy, although this hypothesis requires further investigation.

This study has several limitations. First, this was a retrospective single-institution study with a relatively small cohort, which may limit the generalizability of our findings and the robustness of the observed associations. Second, because pulmonary function testing was not uniformly performed in all patients, the analysis was restricted to patients with available spirometric data for objective classification of COPD status, which may have introduced selection bias. Third, although GOLD classification and emphysema status were evaluated, detailed assessment of COPD phenotypes, including emphysema severity, symptom burden, and exacerbation history, was not uniformly available, which may have contributed to clinical heterogeneity within the COPD group. Finally, although IPTW improved covariate balance, residual and unmeasured confounding cannot be excluded given the observational design and limited sample size.

## Conclusions

COPD may be associated with shorter PFS and OS in ever-smokers with advanced NSCLC receiving first-line ICI plus chemotherapy. These findings suggest that COPD may have prognostic relevance in this population and highlight the potential impact of underlying pulmonary comorbidity on outcomes in patients receiving ICI plus chemotherapy. Larger prospective studies are needed to confirm these findings and to clarify the clinical implications of COPD in the era of chemoimmunotherapy.

## Supplementary Information

Supplementary Material 1: Supplementary Fig. 1. Covariate balance before and after IPTW adjustment in the sensitivity analysis. Standardized mean differences (SMDs) for baseline covariates before and after inverse probability of treatment weighting (IPTW) in the sensitivity analysis. Emphysema status and driver alteration status were added to the primary propensity score model. The dashed line indicates an absolute SMD of 0.10. Abbreviations: COPD, chronic obstructive pulmonary disease; IPTW, inverse probability of treatment weighting; PD-L1, programmed death-ligand 1; ECOG PS, Eastern Cooperative Oncology Group performance status; TPS, tumor proportion score; SMD, standardized mean difference. Supplementary Fig. 2. Progression-free survival and overall survival in the sensitivity analysis. Kaplan–Meier curves for progression-free survival (PFS) (A) and overall survival (OS) (B) in the COPD and non-COPD groups after inverse probability of treatment weighting (IPTW) adjustment. Emphysema status and driver alteration status were added to the primary propensity score model in the sensitivity analysis. Abbreviations: CI, confidence interval; COPD, chronic obstructive pulmonary disease; HR, hazard ratio; IPTW, inverse probability of treatment weighting; NR, not reached; OS, overall survival; PFS, progression-free survival.

Supplementary Material 2: Supplementary Table 1. Adverse events and treatment modifications. Abbreviations: COPD, chronic obstructive pulmonary disease; AE, adverse event. Supplementary Table 2. Covariate balance before and after IPTW in the sensitivity model. Abbreviations: IPTW, inverse probability of treatment weighting; PD-L1, programmed death-ligand 1; TPS, tumor proportion score.

## Data Availability

The datasets used and/or analyzed during the current study are available from the corresponding author upon reasonable request.

## Abbreviations

COPD: Chronic obstructive pulmonary disease
NSCLC: Non-small cell lung cancer
ICIs: Immune checkpoint inhibitors
PD-1: Programmed death-1
PFS: Progression-free survival
OS: Overall survival
IPTW: Inverse probability of treatment weighting
PFT: Pulmonary function test
FEV_1_/FVC: One second to forced vital capacity ratio
GOLD: Global Initiative for Chronic Obstructive Lung Disease
TPS: Tumor proportion score
ECOG PS: Eastern Cooperative Oncology Group performance status
CRP: C-reactive protein
NLR: Neutrophil-to-lymphocyte ratio
BMI: Body mass index
HR: Hazard ratio
CI: Confidence interval
SMD: Standardized mean differences
