# Supplementary material for: COPD is associated with poorer survival in patients with advanced non-small cell lung cancer treated with first-line ICI plus chemotherapy
Source: BMC Pulm Med. 2026 Jul 20;26:472. doi: 10.1186/s12890-026-04509-3 (PMC13663149; doi:10.1186/s12890-026-04509-3)
Supplement: Supplementary file 1 [file 12890_2026_4509_MOESM2_ESM.docx]

**Supplementary table 1. Adverse events and treatment modifications**

|  | **Unadjusted** | |  |
| --- | --- | --- | --- |
|  | **COPD** | **Non-COPD** | **P** |
|  | **(n=33)** | **(n=22)** |  |
| Clinically relevant AEs (%) |  |  |  |
| Febrile neutropenia | 3 (9.1) | 4 (18.2) | 0.419 |
| Pneumonia (Grade ≥2) | 9 (27.3) | 2 (9.1) | 0.168 |
| Other infections | 4 (12.1) | 1 (4.5) | 0.638 |
| Treatment modifications (%) |  |  |  |
| Dose reduction | 11 (33.3) | 11 (50.0) | 0.267 |
| Delay or omission | 4 (12.1) | 4 (18.2) | 0.700 |
| Discontinuation | 4 (12.1) | 0 (0.0) | 0.141 |

Abbreviations: COPD, chronic obstructive pulmonary disease; AE, adverse event.

**Supplementary table 2.** **Covariate balance before and after IPTW in the sensitivity model**

|  | Unadjusted | IPTW adjusted |
| --- | --- | --- |
|  | SMD | SMD |
| Age | 0.441 | 0.051 |
| Sex, male | 0.231 | 0.054 |
| Pack-year | 0.170 | 0.161 |
| Histology, Squamous cell carcinoma | 0.168 | 0.076 |
| PD-L1 TPS <1 | 0.027 | 0.024 |
| PD-L1 TPS 1–49 | 0.095 | 0.000 |
| PD-L1 TPS 50–100 | 0.122 | 0.025 |
| Performance status 0 | 0.071 | 0.078 |
| Driver alteration status | 0.114 | 0.022 |
| Emphysema | 0.207 | 0.060 |

Abbreviations: IPTW, inverse probability of treatment weighting; PD-L1, programmed death-ligand 1; TPS, tumor proportion score.
